# The Validation of an Artificial Intelligence-Based Software for the Detection and Numbering of Primary Teeth on Panoramic Radiographs

**DOI:** 10.3390/diagnostics15121489

**Published:** 2025-06-11

**Authors:** Heba H. Bakhsh, Dur Alomair, Nada Ahmed AlShehri, Alia U. Alturki, Eman Allam, Sara M. ElKhateeb

**Affiliations:** 1 Department of Preventive Dental Sciences, College of Dentistry, Princess Nourah bint Abdulrahman University, P.O. Box 84428, Riyadh 11671, Saudi Arabia; hhbakhsh@pnu.edu.sa (H.H.B.);; 2 Department of Basic Dental Sciences, College of Dentistry, Princess Nourah bint Abdulrahman University, P.O. Box 84428, Riyadh 11671, Saudi Arabia; aualturki@pnu.edu.sa; 3 Research and Graduate Studies Department, Mohammed Bin Rashid University of Medicine and Health Sciences, Dubai P.O. Box 505055, United Arab Emirates

**Keywords:** primary teeth, panoramic radiography, artificial intelligence, sensitivity and specificity

## Abstract

**Background**: Dental radiographs play a crucial role in diagnosis and treatment planning. With the rise in digital imaging, there is growing interest in leveraging artificial intelligence (AI) to support clinical decision-making. AI technologies can enhance diagnostic accuracy by automating tasks like identifying and locating dental structures. The aim of the current study was to assess and validate the accuracy of an AI-powered application in the detection and numbering of primary teeth on panoramic radiographs. **Methods**: This study examined 598 archived panoramic radiographs of subjects aged 4–14 years old. Images with poor diagnostic quality were excluded. Three experienced clinicians independently assessed each image to establish the ground truth for primary teeth identification. The same radiographs were then evaluated using EM2AI, an AI-based diagnostic software for the automatic detection and numbering of primary teeth. The AI’s performance was assessed by comparing its output to the ground truth using sensitivity, specificity, predictive values, accuracy, and the Kappa coefficient. **Results**: EM2AI demonstrated high overall performance in detecting and numbering primary teeth in mixed dentition, with an accuracy of 0.98, a sensitivity of 0.97, a specificity of 0.99, and a Kappa coefficient of 0.96. Detection accuracy for individual teeth ranged from 0.96 to 0.99. The highest sensitivity (0.99) was observed in detecting upper right canines and primary molars, while the lowest sensitivity (0.79–0.85) occurred in detecting lower incisors and the upper left first molar. **Conclusions**: The AI module demonstrated high accuracy in the automatic detection of primary teeth presence and numbering in panoramic images, with performance metrics exceeding 90%. With further validation, such systems could support automated dental charting, improve electronic dental records, and aid clinical decision-making.

## 1. Introduction

The term artificial intelligence (AI) describes a machine’s capacity to carry out intellectual and cognitive tasks that humans can [1]. To accomplish a particular goal, like identifying objects on images or radiographs, most AI applications use machine learning, which continually analyses a training dataset to find and learn innate patterns or predict measures from numerical data. The performance of an AI application is ideally evaluated on unseen test datasets [2,3,4].

The recent literature suggests that AI can improve patient outcomes, increase diagnosis accuracy, and expedite workflows in healthcare settings. In dentistry, many AI applications have been developed, investigated, implemented, and clinically integrated. Dental image analysis using AI has been particularly described as convenient and practical, with diagnostic accuracies similar or superior to those of expert practitioners in certain applications such as detecting oral mucosal lesions, dental implant types, and locations; predicting dental caries; and locating cephalometric landmarks [5,6,7,8].

In pediatric dentistry, AI was proposed for diagnosing conditions such as supernumerary and submerged teeth, early dental caries, and dental plaque. It was also a helpful aid in assessing bone age and developing preventive oral healthcare strategies. AI’s ability to predict outcomes accurately was comparable to experienced pediatric dentists, making it a potential valuable tool in this specialty [9,10]. From patients’ perspective, patients generally had a positive attitude towards AI in dentistry, recognizing its usefulness in diagnostics. The literature reported that AI-supported diagnostics improved the communication of radiographic findings and enhanced patients’ ability to recognize dental issues. However, AI-based communication did not significantly affect patients’ trust in dentists’ diagnoses [11,12,13].

AI currently offers some benefits in pediatric and orthodontic dentistry by facilitating clinical decision-making as well as practice efficiency. Clinically, AI supports the diagnosis and treatment planning process through advanced applications including radiograph interpretation, growth prediction, eruption pattern monitoring, and caries risk assessments. One of the key advantages in pediatric orthodontics is the early detection of dental anomalies such as hypodontia and supernumerary teeth, particularly during the mixed dentition stage [14]. The timely identification of these anomalies is critical for optimal treatment planning, allowing early interventions such as extraction of supernumerary teeth or the strategic preservation of primary teeth to guide eruption and reduce the complexity of future orthodontic procedures. In high-volume pediatric practices, less experienced clinicians may overlook such anomalies on panoramic radiographs. Deep learning models have demonstrated superior speed and accuracy compared to practitioners in detecting mesiodens, as reported by Ahn et al., highlighting AI’s potential to significantly aid clinicians with limited experience and ultimately contribute to more personalized and timely dental care [15,16].

A recently issued policy statement adopted by the FDI World General Federation General Assembly stated that dental AI needs to be useful, true, and built on data of high quality to prevent bias and performance attrition due to limited generalizability. To access high-quality data, a balance needs to be developed between data protection and accessibility for wide utilization. Ensuring that AI adoption will help reduce inequity is closely linked to the underlying training data reflecting diverse populations, as well as the accessibility of such AI to all population groups [2]. The policy also stressed on the significant potential risk of automation bias, which is defined as the over-reliance of practitioners on AI use. Despite all the efforts and developments, attempts to measure the newly introduced AI application’s performance based on standardized datasets using comparable metrics are currently lacking.

Detecting primary teeth on panoramic X-rays is crucial for assessing dental development stages and identifying eruption anomalies. Identifying abnormalities in mixed dentition, such as delayed root resorption, ectopic eruption, and tooth impactions, is essential for the accurate diagnosis and planning of pediatric dentistry and orthodontic interventions that support optimal arch development and facial growth [17]. The aim of the current study was to assess and validate the accuracy of the EM2AI application in the detection and numbering of primary teeth on panoramic radiographs.

## 2. Materials and Methods

Panoramic radiographs originally utilized for several diagnostic purposes were collected from multiple governmental dental outpatient facilities in Riyadh, Saudi Arabia. A total of 630 anonymized digital panoramic radiographs taken for subjects aged 4 to 14 years old were initially obtained. Image acquisition was mostly performed using the Planmeca ProMax 2D imaging unit (Planmeca Oy, Helsinki, Finland) in panoramic mode with pediatric settings. Images with severe distortion or poor quality that rendered individual tooth identification impossible were excluded (*n* = 32). Consequently, 598 diagnostically acceptable images were included for the analysis. The study was approved by the Institutional Review Board (IRB) at Princess Nourah bint Abdulrahman University (IRB number: 21-0164).

**Figure 1:**
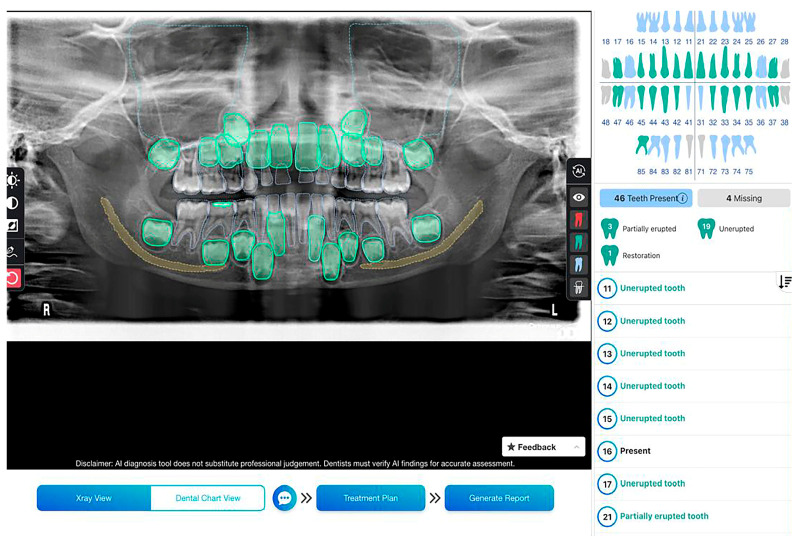
Panoramic radiograph of a 6-year-old subject showing mixed dentition analyzed using EM2AI for primary teeth detection.

To establish the “ground truth”, three calibrated orthodontists, each with over 10 years of experience, independently reviewed the included radiographs to determine the presence or absence of primary teeth in each image using an electronic form. The same images were then processed using EM2AI (EM2AI Version 3.2.0, Clementi, Singapore), a commercially available software as a service (SaaS) platform designed for AI-based dental diagnostics. The EM2AI platform offers web-based support for detecting, annotating, and numbering teeth, including both pathological and non-pathological features. It can also generate automated dental charting and treatment plan suggestions based on AI analysis (Figure 1).

For the specific purpose of this study, the primary teeth detection and numbering function of the EM2AI software was evaluated. The software utilizes an instance segmentation model, specifically the Mask R-CNN architecture. Mask R-CNN is a flexible and advanced convolutional neural network (CNN)-based framework that extends the Faster R-CNN model by incorporating a parallel branch that predicts a segmentation mask for each detected object, in addition to the bounding box. This enables the model to detect both the presence and location of objects in an image, as well as generate segmentation masks outlining each object. This capability makes it particularly suitable for segmenting individual teeth in dental radiographs.

### Statistical Analysis

For the interexaminer reliability assessment of the orthodontic specialists, 40 radiographs were randomly selected from the whole sample and independently evaluated by three investigators by comparing 800 records of primary teeth. Interexaminer reliability was determined using the intraclass correlation coefficient (ICC), which demonstrated a high degree of agreement among the evaluators (ICC = 0.89 and 95% CI: 0.82–0.94).

Data was tabulated and subjected to statistical analysis using IBM SPSS Statistics version 26 (SPSS, Chicago, IL, USA). The performance and accuracy of the AI-based automatic detection for teeth presence and numbering in primary dentition was evaluated relative to the ground truth based on the reporting sensitivity (S), specificity (E), positive predictive value (PPV), negative predictive value (NPV), test accuracy, and Kappa coefficient of agreement (Table 1). AI detection accuracy was calculated based on the following equation:

Accuracy (A) = TP + TN/TP + TN + FP + FN.

## 3. Results

### 3.1. Upper Primary Anterior Teeth

The accuracy of EM2AI in the detection of upper anterior primary teeth was 0.98 for all teeth, except for the upper right lateral incisor, and for the upper left canine, it was 0.97. The highest (0.99) EM2AI sensitivity was in the detection of the upper right canine, and the lowest sensitivity (0.89) was in the detection of the upper right central incisor (Table 2).

### 3.2. Upper Primary Molars

The highest accuracy of EM2AI was reported for the detection of the upper right second primary molar (0.99) followed by 0.98 for the detection of the remaining primary molars. The highest (0.99) EM2AI sensitivity was in the detection of the upper right second primary molar, and the lowest sensitivity (0.97) was in the detection of the upper left first primary molar (Table 3).

### 3.3. Lower Primary Anterior Teeth

The highest accuracy of EM2AI was reported for the detection of lower right and left primary central incisors (0.99), while the least accurate was 0.96 for lower right lateral incisor detection. The highest (0.97) EM2AI sensitivity was in the detection of the lower right and left primary canines, and the lowest sensitivity (0.83) was in the detection of the lower left central incisor and lower right lateral incisor (Table 4).

### 3.4. Lower Primary Molars

The highest accuracy of EM2AI was reported for the detection of the lower right second primary molar (0.99), while the lowest accuracy was 0.97 for lower left first primary molar detection. The highest (0.99) EM2AI sensitivity was in the detection of the lower right first and second primary molars, and the lowest sensitivity (0.97) was in the detection of the lower left first primary molar (Table 5).

### 3.5. Overall Evaluation of Primary Teeth Detection

The overall accuracy of EM2AI for the detection of all primary teeth in mixed dentition compared to the ground truth was 0.98, with a sensitivity of 0.97, a specificity of 0.99, a PPV of 0.99, and an NPV of 0.97. The EM2AI showed high specificity (0.99), indicating a strong ability to accurately identify missing primary teeth, and high sensitivity (0.97), indicating a strong ability to accurately identify present primary teeth. The highest sensitivity of EM2AI (0.98) was in the detection of upper primary canines and upper and lower primary molars. While the lowest sensitivity (0.85) was in the detection of lower primary incisors. The average accuracy of EM2AI was 0.98 in the detection of all primary teeth except for lower primary canines (0.97) (Table 6).

**Figure 2:**
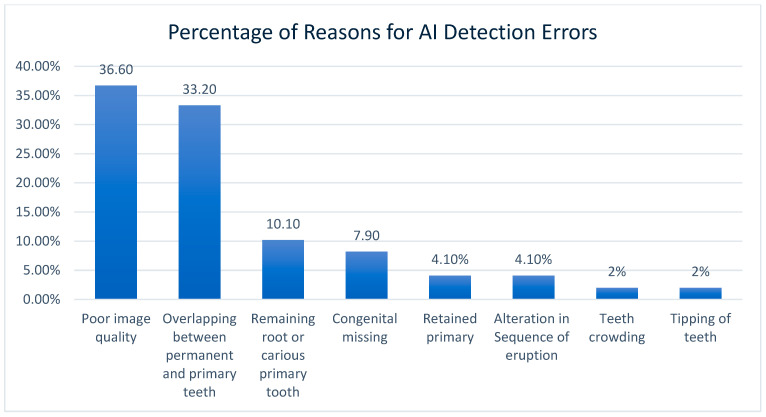
Percentage of reasons for AI detection errors in primary teeth identification among the representative sample.

**Figure 3:**
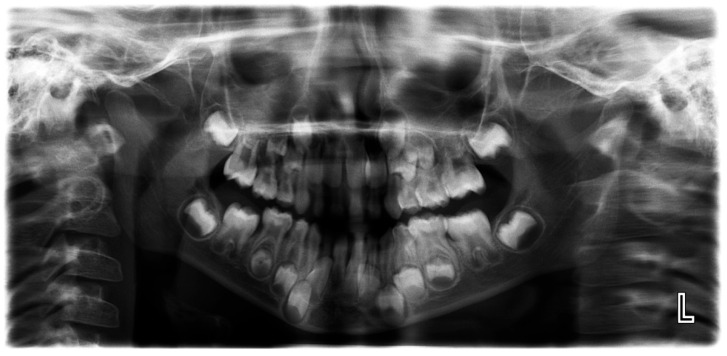
Poor quality image affecting AI performance in the detection of maxillary primary teeth.

**Figure 4:**
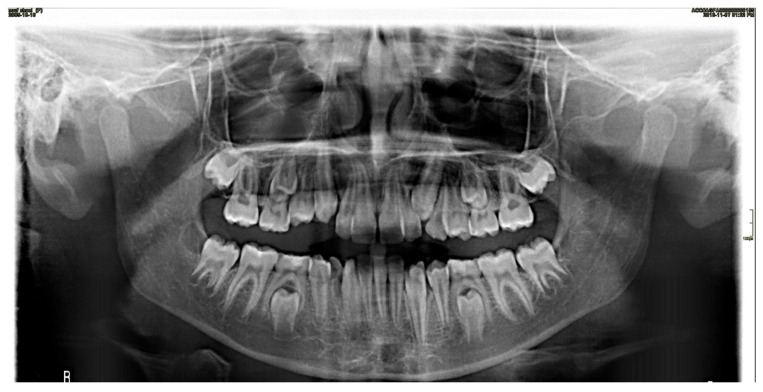
Overlap between the lower left primary and permanent canines affecting the performance of AI in detecting the lower left primary canine.

A random sample of 54 panoramic images, covering all primary teeth across the four quadrants, was selected based on detection errors identified between the AI analysis and the ground truth. An orthodontist and a radiologist reviewed these images to investigate the causes of these discrepancies. Further investigation revealed that 5 of the 54 randomly selected errors were due to inaccuracies in the ground truth. The remaining 49 AI errors in primary teeth identification and numbering were primarily caused by poor image quality (including dark images from excessive exposure, blurring, and magnification due to patient positioning errors, tongue interference, and improper image size selection), overlap between the crowns of permanent and primary teeth, and the missed detection of residual roots of primary teeth. Less common causes included retained primary teeth, altered eruption sequences, the presence of appliances such as space maintainers, unusual tooth positions and angulations, and dental crowding (Figure 2, Figure 3 and Figure 4).

## 4. Discussion

Radiography plays a crucial role in diagnostics and treatment planning in modern dentistry. It is considered the main resource available for clinicians to examine dental anatomy, pathological conditions, and assess treatment outcomes. Dental panoramic radiography is one of the most commonly utilized techniques among the various imaging modalities. For general oral examinations, panoramic radiography is appropriate given with minimal irradiation doses and amount of information provided. As an extraoral imaging method, it provides a comprehensive view of the dental arches, dentition, temporomandibular joints, and maxillary sinuses, making it invaluable across multiple dental specialties. In pediatric dentistry and orthodontics, it has several applications, including the detection of dental anomalies, the assessment of tooth position and development, and the monitoring of treatment progress [18,19,20]. The aim of the current study was to assess and validate the accuracy of a new AI application in the detection and numbering of primary teeth on panoramic radiographs.

AI is increasingly being integrated into dentistry, offering transformative potential in diagnosis, treatment planning, and patient management. This integration aims to enhance the accuracy and efficiency of dental care, providing significant benefits across various dental specialties. AI technologies are currently being utilized across multiple dental specialties, including dental public health, endodontics, oral and maxillofacial surgery, orthodontics, pediatric dentistry, periodontics, and prosthodontics. These technologies assist in diagnosis, clinical decision-making, treatment planning, and prognosis prediction, offering some appreciated insights that enhance the decision-making processes of dental professionals [21,22,23].

AI also plays a crucial role in dental imaging. Studies indicated usefulness in the detection of oral features such different types of fillings, caries, and implants. Its ability to analyze large volumes of data quickly enhances its diagnostic capabilities and efficiency with expected benefits leading to better patient outcomes [24,25]. AI application helped predict failures in clinical scenarios and offered reliable solutions according to recent reports. The dental community expects these applications to make dental care more efficient and economical in the future [26,27].

Although most of the previous reports highlight the promising potential of AI applications in dentistry, particularly in diagnostics, for enhancing patient management and improving the quality of care through increased diagnostic precision and treatment accuracy, several challenges persist. These challenges include data availability, data uniformity, and computational power, especially for handling 3D data. Additionally, the need for considerably high-quality data, potential algorithmic bias, a lack of contextual understanding, and ethical concerns are considered serious limitations. Therefore, AI continues to be regarded as a complementary tool that has the potential to support clinical decision-making, enhance diagnostic accuracy, and improve workflow efficiency rather than a replacement for the expertise, critical thinking, and patient-centered judgment of trained dental practitioners [28,29,30].

The results of the current study indicated that the overall accuracy of the tested AI application for the detection of all primary teeth in mixed dentition compared to the ground truth was 98%, in addition to high specificity (99%), indicating a strong ability to accurately identify missing primary teeth and high sensitivity (97%) and indicating a strong ability to accurately identify present primary teeth. The highest sensitivity was in the detection of upper primary canines, followed by upper and lower primary molars, while the lowest sensitivity was in the detection of lower primary incisors. In agreement with these findings, a recent study assessed a similar AI-driven tool and reported high accuracy and excellent performance in tooth detection (98.9% sensitivity and 99.6% precision) and segmentation [31]. It was also reported that the method utilized significantly reduced the manual segmentation time by 67%, highlighting its efficiency [31].

According to the study by Turosz et al., another AI-driven software demonstrated high accuracy (>90%) in detecting missing teeth, root canal fillings, and implant abutment crowns from dental panoramic radiographs, while its sensitivity and precision were moderate for endodontic lesions [32]. Likewise, a study by Gunec et al. reported that AI applications generated more accurate diagnoses compared to those of junior practitioners in detecting periapical lesions on panoramic radiographs [33]. It was therefore suggested that this form of applications is a convenient and cost-effective screening tool that could potentially reduce the likelihood of introducing human errors due to examiner fatigue. It is, however, important to exemplify that while AI shows promise as a diagnostic aid, clinician verification remains essential, particularly in more complex cases.

In the current study, as the data was being collected, the investigators had several procedural observations. AI exhibited several matching errors including the inability to identify primary teeth in some radiographs due to overlapping between the crowns of permanent and primary teeth. AI struggled to detect primary teeth in poor-quality panoramic radiographs due to either dark images, blurring, magnification, tongue spaces, or poor image size selection. AI missed the detection of the remaining roots of primary teeth. AI had difficulty in recognizing retained primary teeth. The altered sequence of teeth eruption, the presence of appliances like a space maintainer, the unusual position and angulation of the teeth, and crowding led to misidentification by AI.

It is essential that the research community supports clinicians with data and evidence to allow them to test and fully trust the proposed AI-driven models for a number of key applications including the classification of primary teeth from pediatric panoramic radiographs. By leveraging convolutional neural network (CNN)-based architectures, it will be possible to highlight AI’s potential in enhancing diagnostic efficiency while reducing clinician workload. This information is also required to reinforce the role of AI in dentistry, offering promising advancements in automated classification and decision support. In a study by Jaiswal et al., a newly introduced AI application outperformed other CNN-based models in classifying primary teeth, achieving 98% accuracy, precision, recall, and F1 scores. The study utilized a dataset of 620 panoramic radiographs with a 70:15:15 split for training, validation, and testing [34]. These observations, together with the current study findings, suggest that AI-driven classification can enhance diagnostic accuracy, streamline decision-making, and reduce clinician workload in orthodontics and pediatric dentistry applications.

This study has several limitations that should be acknowledged. The age range of the included subjects was broad, which may have introduced uncontrolled variability. This could affect the generalizability of the findings to specific age groups. The dataset was also limited to a specific population and geographic location, and the performance of the EM2AI application may vary in different demographic or clinical settings. Finally, the retrospective nature of the study and reliance on existing panoramic radiographs may have limited control over image quality and standardization. Future studies should consider prospective designs, age-specific subgroup analyses, and multi-center data to enhance the robustness and generalizability of AI-based dental diagnostics.

## 5. Conclusions

In conclusion, the AI module demonstrated high accuracy in the automatic detection of primary teeth presence and numbering in panoramic images, with performance metrics exceeding 90%. Once validated through more extensive research and reporting, it could provide an innovation that enables the automatic preparation of dental charts and electronic dental records and supports clinical decision-making. Critical appraisal of the evidence supporting the use of the different AI applications requires further validation, including the assessment of its relevance and usefulness in various target contexts. Further studies are recommended to allow for the testing of the AI model in identifying and numbering permanent teeth and in diagnosing developmental and dental anomalies such as submerged primary teeth, supernumerary teeth, ectopic eruption, and impactions.

## Figures and Tables

**Table 1:** Definitions of the AI performance metrics.

Sensitivity	The proportion of the testing method’s true positive results to the reference method’s all positive results.
Specificity	The proportion of the testing method’s true negative results to the reference method’s all negative results.
Positive Predictive Value (PPV)	The proportion of positive test results that are actually positive according to the reference method to total test positive results.
Negative Predictive Value (NPV)	The proportion of negative test results that are actually negative according to the reference method to total test negative results.
Test accuracy	The proportion of the number of true test results to all test results.
Kappa coefficient of agreement	Measures the degree of agreement accounting for the fact that the two methods may happen to agree on some cases by pure chance.

The 95% confidence intervals of all variables were calculated and reported.

**Table 2:** AI performance against the ground truth in the detection of upper primary anterior teeth in mixed dentition.

Tooth Number	Sensitivity	Specificity	PPV	NPV	Accuracy
53	0.99	0.97	0.99	0.95	0.98
52	0.91	0.99	0.98	0.97	0.97
51	0.89	0.99	0.94	0.98	0.98
61	0.93	0.99	0.95	0.99	0.98
62	0.93	0.99	0.97	0.98	0.98
63	0.97	0.98	0.99	0.90	0.97

AI: artificial intelligence, PPV: positive predictive value, NPV: negative predictive value.

**Table 3:** AI performance against the ground truth in the detection of upper primary molars in mixed dentition.

Tooth Number	Sensitivity	Specificity	PPV	NPV	Accuracy
55	0.99	0.98	1.00	0.96	0.99
54	0.98	0.97	0.98	0.97	0.98
64	0.97	0.99	0.99	0.96	0.98
65	0.98	0.98	0.99	0.94	0.98

AI: artificial intelligence, PPV: positive predictive value, NPV: negative predictive value.

**Table 4:** AI performance against the ground truth in the detection of lower primary anterior teeth in mixed dentition.

Tooth Number	Sensitivity	Specificity	PPV	NPV	Accuracy
73	0.97	0.98	0.99	0.94	0.97
72	0.88	0.99	0.97	0.98	0.98
71	0.83	1.00	0.91	0.99	0.99
81	0.87	0.99	0.87	0.99	0.99
82	0.83	0.99	0.94	0.97	0.96
83	0.97	0.96	0.98	0.95	0.97

AI: artificial intelligence, PPV: positive predictive value, NPV: negative predictive value.

**Table 5:** AI performance against the ground truth in the detection of lower primary molars in mixed dentition.

Tooth Number	Sensitivity	Specificity	PPV	NPV	Accuracy
75	0.98	0.98	0.99	0.96	0.98
74	0.97	0.98	0.99	0.95	0.97
84	0.99	0.98	0.99	0.98	0.98
85	0.99	1.00	1.00	0.96	0.99

AI: artificial intelligence, PPV: positive predictive value, NPV: negative predictive value.

**Table 6:** AI performance against the ground truth in the detection of primary teeth in mixed dentition (overall evaluation).

Tooth Number	Sensitivity	Specificity	PPV	NPV	Accuracy
Upper primary incisors	0.92	0.99	0.97	0.98	0.98
Upper primary canines	0.98	0.97	0.99	0.92	0.98
Upper primary molars	0.98	0.98	0.99	0.96	0.98
Lower primary incisors	0.85	0.99	0.94	0.98	0.98
Lower primary canines	0.97	0.98	0.99	0.94	0.97
Lower primary molars	0.98	0.98	0.99	0.96	0.98
All primary teeth	0.97	0.99	0.99	0.97	0.98

AI: artificial intelligence, PPV: positive predictive value, NPV: negative predictive value.

## Data Availability

The original contributions presented in this study are included in the article. Further inquiries can be directed to the corresponding author.
